# Spiritual Care Practices and Competence Among Critical Care Nurses in Türkiye: A Structural Equation Modeling Approach

**DOI:** 10.1007/s10943-025-02353-z

**Published:** 2025-06-03

**Authors:** Öznur Erbay Dallı

**Affiliations:** Department of Internal Medicine Nursing, Bursa Uludag University Faculty of Health Sciences, 16059 Nilüfer, Bursa Türkiye

**Keywords:** Spiritual care, Critical care nurses, Competence, Intensive care

## Abstract

This study aimed to examine the relationship between spiritual care (SC) practices and competence among critical care nurses (CCNs) in Türkiye using structural equation modeling (SEM). A total of 323 CCNs participated, and data were collected using the Nurse Information Form, Nurse Spiritual Care Therapeutics Scale (NSCTS) and Spiritual Care Competence Scale (SCCS). The mean NSCTS and SCCS total scores were 46.53 ± 9.57 and 77.31 ± 12.46 points, respectively. A significant positive correlation was found between SCCS and NSCTS scores (r = 0.712, *p* < 0.01). SEM analysis showed that all SCCS subdimensions significantly predicted SC practices, with professionalization and patient counseling (β = 0.393, *p* = 0.001) having the strongest effect, followed by attitude towards patient spirituality (β = 0.232, *p* = 0.026) and assessment and implementation (β = 0.171, *p* = 0.006). The model demonstrated good fit indices (χ^2^/df = 3.121; GFI = 0.925; CFI = 0.938; RMSEA = 0.069) and accounted for 54.5% of the variance in SC practices. These findings highlight the need for structured education, interdisciplinary collaboration, and institutional strategies to enhance SC integration in intensive care units.

## Introduction

Providing spiritual care is a fundamental aspect of holistic nursing, helping patients find meaning, comfort, and emotional support in times of illness (Timmins & Caldeira, 2017). In intensive care units (ICUs), where patients often face life-threatening conditions and existential distress, spiritual care becomes even more critical (Klimasiński, 2021). Critical care nurses (CCNs), who spend the most time with patients, are in a unique position to address their spiritual needs by offering emotional reassurance, facilitating communication about personal beliefs, and providing support during moments of uncertainty (Dural, 2024; Willemse et al., 2020). However, despite its recognized importance, spiritual care remains inconsistently integrated into ICU nursing practice, largely due to the urgency of physiological interventions, time constraints, lack of knowledge and awareness, and a lack of structured institutional support (Alch et al., 2021; Harrad et al., 2019; Willemse et al., 2018).

The ICU environment poses distinct challenges to spiritual care delivery, as nurses are frequently required to prioritize acute medical needs over holistic care (Abu-El-Noor, 2016). Many ICU patients are sedated, intubated, or unable to communicate, making direct engagement in spiritual discussions difficult (Berning et al., 2016; Dalle Ave & Sulmasy, 2025). Additionally, high patient acuity, workload pressures, and the absence of dedicated spiritual care professionals, such as chaplains or psychologists, further restrict nurses’ ability to provide spiritual care in intensive care settings (Bone et al., 2018; Çınar & Aslan, 2017; Meeprasertsagool et al., 2025).

Although the relationship between spiritual care practices and spiritual care competence has been explored in various healthcare settings (Kurtgöz et al., 2024; Vogel & Schep-Akkerman, 2018), research specifically focusing on CCNs remains scarce. The extant literature often examines these concepts separately, failing to provide a comprehensive analysis of how CCNs’ spiritual care competence influences their engagement in spiritual care interventions in the ICU context. Understanding the factors that shape CCNs’ engagement in spiritual care is essential for identifying gaps in practice, training needs, and institutional policies that may support or hinder spiritual care delivery.

This study aimed to examine the relationship between spiritual care practices and spiritual care competence among CCNs in Türkiye and identify the factors influencing this relationship using structural equation modeling (SEM).

## Methods

### Study Design

This study was a cross-sectional design implemented through a web-based survey. The Checklist for Reporting Results of Internet E-Surveys (CHERRIES) was utilized to guide reporting in this study.

### Sample, Setting and Recruitment

A web-based survey was used to invite CCNs who follow *Nurse Turkey*, a social media platform dedicated to nursing information and support in Türkiye, to participate in the study. After obtaining ethical approval, the researcher contacted the platform administrator and requested the distribution of the survey link through the platform’s social media channels to reach potential participant. To maximize participation, a convenience sampling approach was adopted. The eligibility criteria included: (a) voluntarily agreeing to complete the online survey and (b) having at least one year of experience working in the ICU of a hospital.

The study was conducted with 323 nurses. A post hoc power analysis was performed using G*Power 3.1.9.7 software, considering a confidence level of 95% (α = 0.05), an effect size of 0.30, and a total sample size of 323. The analysis indicated a statistical power of 99%, confirming the adequacy of the sample size for a two-tailed hypothesis test.

### Data Collection Procedures and İnstruments

Following ethical approval, the data collection tools were transferred to the Google Forms platform, which was structured to prevent multiple submissions from the same respondent. The survey link was then shared with the relevant social media platform, and CCNs were invited to participate. The principal investigator did not have direct contact with the CCNs and ensured adherence to data confidentiality regulations.

The first section of the online survey included a consent page outlining the study’s purpose and significance, the eligibility requirements, the names of the questionnaires used, the estimated time required to complete the survey, details on data storage policies, confidentiality assurances, and the contact information of the principal investigator. Participants were also informed that they could discontinue their participation at any point. Only those who selected "Yes" in response to the question "Do you voluntarily consent to participate in this study?" were granted access to the subsequent sections of the survey, whereas those who selected "No" were unable to proceed further.

To prevent missing data, all questions were marked as mandatory on the Google Forms platform, ensuring that participants responded to every item. The survey remained open for responses for 28 days, from December 15, 2024, to January 12, 2025. Only fully completed surveys were included in the final analysis. No additional strategies, such as financial incentives, lottery draws, rewards, or reminder postcards, were implemented to encourage participation. The Google Form’s timer function indicated that the survey required between 8 and 14 min to complete.

The following tools were utilized for data collection: (a) the Nurse Information Form (NIF), (b) the Nurse Spiritual Care Therapeutics Scale (NSCTS), and (c) the Spiritual Care Competence Scale (SCCS).

### Nurse Information Form (NIF)

This form was designed by the researcher based on relevant literature to gather data on CCNs’ sociodemographic (e.g., age, gender), professional (e.g., current unit, work experience), and spiritual care characteristics (e.g., barriers to providing spiritual care, previous training on the subject) (Baguna et al., 2024; Bakir et al., 2017; Heidari et al., 2022; Kurtgöz et al., 2024).

### Nurse Spiritual Care Therapeutics Scale (NSCTS)

The NSCTS, developed by Mamier and Taylor (2015) and validated in Turkish by Aslan et al. (2020), assesses how frequently nurses provide spiritual care in clinical settings. It consists of 17 items within a single dimension, rated on a 5-point Likert scale (1 = never, 0 times; 2 = rarely, 1–2 times; 3 = sometimes, 3–6 times; 4 = usually, 7–11 times; 5 = very often, 12 times or more). Total scores range from 17 to 85, with higher scores indicating more frequent engagement in spiritual care. The scale demonstrated strong reliability, with a Cronbach’s Alpha of 0.86 in the Turkish validation study (Aslan et al., 2020) and 0.88 in the present study.

### Spiritual Care Competence Scale (SCCS)

The SCCS evaluates nurses’ competencies in delivering spiritual care and was originally developed by Van Leeuwen et al. (2009), with its Turkish adaptation and psychometric assessment conducted by Daghan et al. (2019). The scale comprises 27 items rated on a 5-point Likert scale and is divided into three subscales: Assessment and Implementation of Spiritual Care (AISC), Professionalization of Spiritual Care and Patient Counseling (PSCPC), and Attitude Towards the Patient’s Spirituality and Communication (ATPSC). Scores range from 27 to 135, with higher scores indicating greater competence in spiritual care. The original study reported a Cronbach’s Alpha of 0.97 (Daghan et al., 2019), while in the present study, it was calculated as 0.84.

### Data Analysis

The study data were analyzed using SPSS (Statistical Package for Social Sciences, version 29.0) and AMOS (Analysis of Moment Structures, version 29) for Windows. Normality was tested using skewness/kurtosis values and the Shapiro–Wilk test, which indicated that most variables followed a normal distribution. Descriptive statistics, including mean, standard deviation, percentage, and frequency, were used to summarize participants’ sociodemographic and other characteristics.

To examine the relationship between CCNS’ spiritual care practices and the competence, Pearson correlation analysis was performed. The strength of correlations was categorized as follows: 0.20–0.40 (low), 0.40–0.70 (moderate), 0.70–0.90 (high), and 0.90–1.00 (very strong). Subsequently, structural equation modeling (SEM) with maximum likelihood estimation was conducted to test the theoretical model. In the hypothesized model, AISC, PSCPC and ATPSC were included as independent variables, while the spiritual care practices provided by nurses was the dependent variable. Model fit was assessed using the following indices: chi-square (χ^2^)/degree of freedom (df) (< 5), goodness-of-fit index (GFI) (> 0.85), comparative fit index (CFI) (> 0.90), and root mean square error of approximation (RMSEA) (< 0.08). Statistical significance was set at *p* < 0.05.

## Results

### Characteristics of the Nurses

The mean age of the 323 CCNs who participated in the study was 33.42 ± 5.26 years (Table 1). The majority of the participants were female (80.2%) and held a bachelor’s degree (75.2%). Most nurses worked in university hospitals (39.3%), followed by public hospitals (27.3%). The largest proportion of participants were from the Marmara (26.7%) and Central Anatolia (23.2%) regions.

**Table 1:** Demographic, professional, and spiritual care-related characteristics of the CCNs (N = 323)

Characteristics	n (%) or M ± SD
Age (years)	33.42 ± 5.26
Gender	
Female	259 (80.2)
Male	64 (19.8)
Education	
Associate degree	31 (9.6)
Bachelor’s degree	243 (75.2)
Postgraduate degree	49 (15.2)
Type of hospital	
University	127 (39.3)
Public	88 (27.3)
City	63 (19.5)
Private	45 (13.9)
Region of employment	
Marmara	86 (26.7)
Aegean	48 (14.8)
Mediterranean	52 (16.1)
Black sea	22 (6.8)
Central anatolia	75 (23.2)
Eastern anatolia	24 (7.4)
Southeastern anatolia	16 (5.0)
Type of ICU ward	
Anesthesiology and reanimation	59 (18.3)
General/mix	67 (20.7)
Medical	48 (14.9)
General surgery	23 (7.1)
Cardiovascular/cardiology	60 (18.6)
Pediatric/newborn	36 (11.1)
Others^a^	30 (9.3)
Work experience in ICU (years)	6.12 ± 2.89
Work experience (categorical)	
1–5 years	198 (61.3)
> 5 years	125 (38.7)
Nurse:patient ratio	
1:2	190 (58.8)
1:3	92 (28.5)
≥ 1:4	41 (12.7)
Presence of SC professional in the unit/hospital (e.g., chaplain, psychologist)	
Yes	34 (10.5)
No	289 (89.5)
Knowledge of SC	
Yes	273 (84.5)
No	50 (15.5)
Receiving training on SC	
Yes	61 (18.9)
No	262 (81.1)
Considering SC to be important for nursing	
Yes	280 (86.7)
No	43 (13.3)
Considering SC as an important need for patients	
Yes	270 (83.6)
No	53 (16.4)
Status of SC providing	
Yes	166 (51.4)
No	157 (48.6)
If yes, types of SC provided^b^	
Active and empathic listening	126 (76.0)
Supportive responding	147 (88.6)
Non-prejudicial approach	112 (67.5)
Respecting patients’ beliefs	139 (83.7)
Providing hope and emotional support	132 (79.5)
Accepting the patient’s feelings	127 (76.5)
Support through touch	79 (47.5)
Helping patients meet their spiritual needs	99 (59.6)
Providing comforting ınterventions (e.g., silence, music, etc.)	76 (45.8)
Providing opportunities for family interaction	52 (31.3)
Supporting patient’s family members	49 (29.5)
Barriers to providing SC^b^	
ICU environment	298 (92.3)
Criticality of the patient	270 (83.6)
Lack of knowledge and awareness	209 (64.6)
Lack of integration of spiritual care into routine practice	305 (94.4)
Lack of a spiritual advisor or support team	252 (78.0)
Lack of time	200 (68.1)
Workload	248 (76.8)
Hospital or unit policies	190 (58.8)

^a^Neurology, neurosurgery, burn, pulmonology ICUs; ^b^multiple answers

Abbreviations: M, mean; SD, standard deviation: ICU, Intensive care unit; SC: Spiritual care

The most common ICU settings were general/mixed ICUs (20.7%), cardiovascular/cardiology ICUs (18.6%), and anesthesiology and reanimation ICUs (18.3%). The mean ICU work experience was 6.12 ± 2.89 years, and more than half of the nurses (58.8%) reported a nurse-to-patient ratio of 1:2 (Table 1).

Most nurses (89.5%) reported the absence of a spiritual care professional in their unit or hospital. A majority of nurses (84.5%) reported having knowledge of spiritual care, yet only 18.9% had received training. Most participants considered spiritual care important for nursing (86.7%) and an essential patient need (83.6%). About half of the CCNs (51.4%) reported providing spiritual care. Among the types of spiritual care provided, the most frequently reported were supportive responding (88.6%), respecting patients’ beliefs (83.7%), providing hope and emotional support (79.5%), active and empathic listening (76.0%), and accepting the patient’s feelings (76.5%). The most commonly identified barriers to providing spiritual care were lack of integration into routine practice (94.4%), ICU environment (92.3%), critical condition of the patient (83.6%), lack of a spiritual advisor or support team (78.0%), and workload (76.8%) (Table 1).

The mean total SCCS score among CCNs was 77.31 ± 12.46 points. Among its subscales, the mean scores were 16.46 ± 3.50 points for AISC, 40.06 ± 7.11 for PSCPC, and 20.63 ± 5.42 points for ATPSC. The mean NSCTS score was calculated as 46.53 ± 9.57 (Table 2).

**Table 2:** Spiritual care practices and competence levels of CCNs (N = 323)

Scales	Range of scores	M ± SD
SCCS		
Assessment and ımplementation of SC	6–30	16.46 ± 3.50
Professionalization of SC and patient counseling	15–75	40.06 ± 7.11
Attitude towards the patient’s spirituality and communication	6–30	20.63 ± 5.42
Total score	27–135	77.31 ± 12.46
NSCTS		
Total score	17–85	46.53 ± 9.57

M: Mean, SD: Standard Deviation, SC: Spiritual Care, NSCTS: Nurse Spiritual Care Therapeutics Scale, SCCS: Spiritual Care Competence Scale

A statistically significant positive high correlation was observed between the total NSCTS and the SCCS score (r = 0.712, *p* < 0.01), as well as its subscales, including AISC (r = 0.602, *p* < 0.01), PSCPC (r = 0.711, *p* < 0.01), and ATPSC (r = 0.714, *p* < 0.01) (Table 3).

**Table 3:** Pearson correlation between CCNs’ spiritual care practices and competence (N = 323)

Variable	SCCS score			
	Total	AISC	PSCPC	ATPSC
Total NSCTS score	0.712*	0.602*	0.711*	0.714*

NSCTS, Nurse Spiritual Care Therapeutics Scale; SCCS, Spiritual Care Competence Scale; AISC, Assessment and Implementation of Spiritual Care; PSCPC, Professionalization of Spiritual Care and Patient Counseling; ATPSC, Attitude Towards the Patient’s Spirituality and Communication

*p < 0.01

### Structural Equation Modeling

**Fig. 1:**
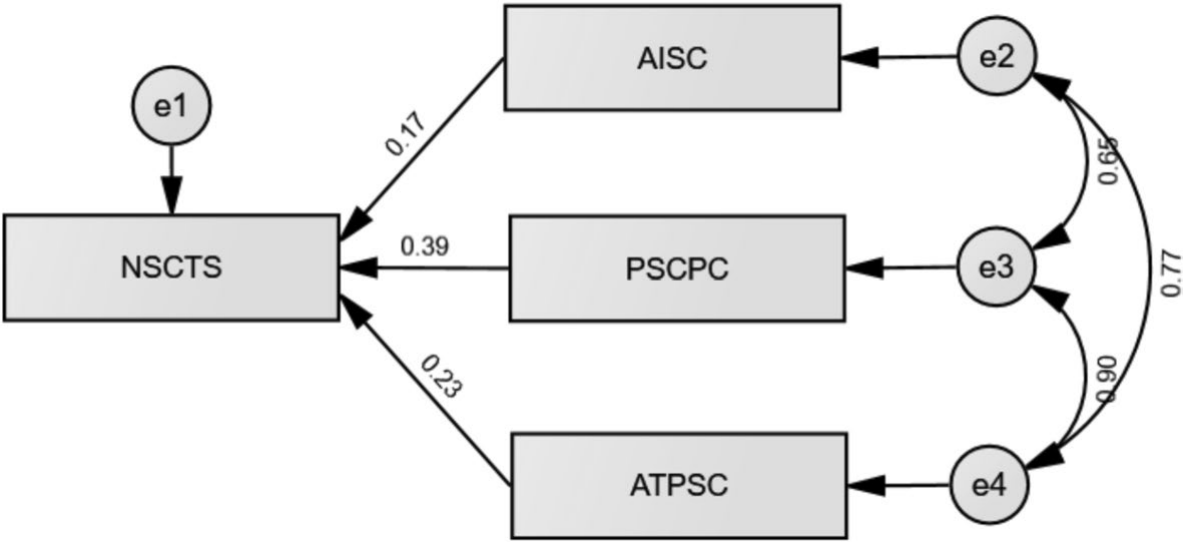
The path diagram of the model

SEM was conducted to examine the influence of spiritual care competence components on spiritual care practices among CCNs (Fig. 1). The model was performed using the maximum likelihood estimation method and demonstrated an acceptable model fit: χ^2^/df = 3.121, GFI = 0.925, CFI = 0.938, RMSEA = 0.069. Path analysis revealed that AISC (β = 0.171, *p* = 0.006), PSCPC (β = 0.393, *p* = 0.001), and ATPSC (β = 0.232, *p* = 0.026) significantly predicted NSCTS scores (Table 4). The independent variables collectively explained 54.5% of the variance in spiritual care practices (R^2^ = 0.545).

**Table 4:** Results of structural equation modeling analysis

Dependent variable	Independent variable	Standardized β	95% CI	*p*-value
NSCTS	AISC	0.171	0.165, 0.962	**0.006**
NSCTS	PSCPC	0.393	0.369, 0.940	**0.001**
NSCTS	ATPSC	0.232	0.047, 0.725	**0.026**

NSCTS, Nurse Spiritual Care Therapeutics Scale; SCCS, Spiritual Care Competence Scale; AISC, Assessment and Implementation of Spiritual Care; PSCPC, Professionalization of Spiritual Care and Patient Counseling; ATPSC, Attitude Towards the Patient’s Spirituality and Communication; CI, Confidence Interval

The bold *p*-values indicate a statistical significance of less than 0.05

Model fit: *x*^2^/df = 3.121; GFI = 0.925; CFI = 0.938; RMSEA = 0.069

*R*^2^ = 0.545

## Discussion

This study provides critical insights into the relationship between spiritual care competence and engagement in spiritual care practices among CCNs. A structural equation modeling approach was employed to identify key predictors of spiritual care provision, demonstrating that spiritual care competence is closely linked to nurses’ ability to engage in holistic patient care within the ICU setting. The SEM results demonstrated that all dimensions of spiritual care competence—assessment and implementation, professionalization and patient counseling, and attitudes towards patient spirituality—were significant predictors of spiritual care practices, suggesting that competence in these areas may play a crucial role in shaping nurses’ ability to address patients’ spiritual needs.

The total SCCS score in this study was 77.31 ± 12.46, indicating a moderate level of spiritual care competence among CCNs. This finding is consistent with previous studies that have reported similar SCCS scores among CCNs (Koroglu & Öksüz, 2024; Mohamed Elsayed et al., 2023). Among the subscales, the PSCPC had the highest mean score, suggesting that CCNs feel more confident in the professional aspects of spiritual care and patient guidance. However, the AISC subscale yielded a lower mean score, indicating potential challenges in identifying and applying spiritual care practices in clinical settings. Similarly, the ATPSC subscale suggests that while CCNs may hold positive views on spiritual care, their ability to effectively integrate it into communication and patient interactions may vary. The NSCTS total score of 46.53 ± 9.57 further reflects a moderate frequency of spiritual care practices, which may be influenced by competence levels and institutional barriers. When compared to a previous study involving palliative and oncology clinical nurses, where the SCCS total score was reported as 98.62 ± 18.08 points, the scores of CCNs in this study appear lower. The same trend is observed across subscales, as nurses scored higher in AISC (20.95 ± 5.20 points), PSCPC (51.94 ± 11.75 points), and ATPSC (25.73 ± 4.35 points) (Kurtgöz et al., 2024). These differences may be attributed to the unique challenges of intensive care settings, where nurses face greater time constraints, higher patient acuity, and a technology-driven environment that may deprioritize spiritual care. Unlike general clinical settings, CCNs must continuously monitor critically ill patients, manage complex medical equipment, and respond to rapid physiological changes, leaving limited opportunities for holistic interventions (Badanta et al., 2022; Bakir et al., 2017; Willemse et al., 2020). Consistently, many nurses in this study identified the ICU environment, patient criticality, and workload as major barriers to providing spiritual care. Similarly, previous scoping review has highlighted that the demanding nature of ICU workflows and the biomedical focus of intensive care often limit nurses’ ability to integrate spiritual care into routine practice (Kappes et al., 2025). Additionally, the lack of integration into routine practice and absence of a spiritual care team further restricted their ability to engage in spiritual care interventions, aligning with literature that points to institutional barriers and the absence of structured support systems as key challenges (Kappes et al., 2025; Momeni et al., 2022).

The correlation analysis revealed a strong positive relationship between spiritual care competence and engagement in spiritual care practices, reinforcing previous research that has identified competency as a key factor in nurses’ ability to integrate spiritual care into their clinical roles (Baguna et al., 2024; Heidari et al., 2022; Kurtgöz et al., 2024; Mohamed Elsayed et al., 2023). All SCCS subscales showed significant correlations with spiritual care practices, suggesting that higher competence in any dimension of spiritual care enhances the likelihood of engaging in spiritual care interventions. Similarly, SEM results further confirmed that all dimensions of spiritual care competence significantly predicted spiritual care practices, with professionalization and patient counseling showing the strongest predictive value. This suggests that spiritual care competence is not only associated with but also a key determinant of the extent to which nurses engage in spiritual care interventions. However, as discussed earlier, barriers such as high workload, the ICU environment, and a lack of institutional support may still limit the full integration of spiritual care into routine nursing practice, even among highly competent CCNs. Supporting spiritual care practices and enhancing competence of nurses is crucial, as it may contribute to improved patient outcomes. A meta-analysis by Li et al. (2024) found that spiritual care interventions in ICU settings such as prayer, relaxation techniques, music therapy, and mindfulness-based spiritual support significantly improved patients’ consciousness levels, reduced anxiety, and enhanced patient comfort, while also contributing to shorter ICU stays and overall well-being​. To overcome barriers to spiritual care practices, integrating spiritual care assessments into routine nursing documentation and care plans could help standardize its practice in ICUs. Strengthening interdisciplinary collaboration with chaplains or psychologists may also alleviate some of the burden on nurses. The Society for Critical Care Medicine emphasizes that chaplains play a crucial role in providing objective crisis intervention and spiritual support for patients and families (Willemse et al., 2020). In this regard, Berning et al. (2016) describe chaplain interventions as a significant step toward integrating spiritual care into daily ICU practice, emphasizing the role of chaplains in supporting both patients and healthcare professionals. Some studies highlight that the integration of a board-certified chaplain into the interdisciplinary team contributes to improved patient satisfaction, emphasizing the value of structured spiritual care within healthcare settings (Hughes et al., 2007; Wall et al., 2007). Workload management strategies, including appropriate staffing and equitable task distribution, may help create more opportunities for holistic nursing care. Additionally, structured training programs and professional development initiatives could enhance nurses’ competence and confidence in providing spiritual care. For instance, Riahi et al. (2018) found that spiritual intelligence training significantly improved CCNs’ spiritual care competence, highlighting the importance of education in overcoming barriers such as lack of knowledge and confidence. The study by Abusafia et al. (2024) found that a two-week structured Nursing Spiritual Care Module, which included theoretical knowledge, case-based discussions, and role-playing exercises, significantly improved nurses’ spiritual care competence.

### Limitations

This study has some limitations. This study’s generalizability is limited due to its specific timing and voluntary participation of Turkish CCNs through the Nurse Turkey social media platform. The reliance on self-reported data introduces the possibility of social desirability bias, as nurses may have provided responses that reflect perceived expectations rather than actual practice. Lastly, cultural and institutional differences in spiritual care practices may limit the applicability of the results to healthcare settings outside of Türkiye. Future studies incorporating longitudinal designs, diverse sampling strategies, and objective patient-centered outcomes could provide deeper insights into the role of spiritual care in intensive care settings.

## Conclusion

This study examined the relationship between spiritual care practices and competence among CCNs in Türkiye, revealing that higher competence was significantly associated with increased engagement in spiritual care interventions. However, systemic barriers such as high workload, the ICU environment, and limited institutional support were identified as major challenges to integrating spiritual care into routine practice. The findings highlight the importance of structured training programs, interdisciplinary collaboration, and the inclusion of spiritual care professionals to support nurses in providing holistic patient care. Strengthening institutional policies and addressing workload-related constraints may further facilitate the integration of spiritual care in ICUs. Future research should explore long-term strategies for enhancing spiritual care implementation and its impact on both patient outcomes and nurse well-being.

## Data Availability

All the data generated or analyzed during this study are included in this published article.

## References

[CR1] Abu-El-Noor, N. (2016). ICU nurses’ perceptions and practice of spiritual care at the end of life: Implications for policy change. *Online Journal of Issues in Nursing,**21*(1), 6. 10.3912/OJIN.Vol21No01PPT05 27853263 10.3912/OJIN.Vol21No01PPT05

[CR2] Abusafia, A. H., Khraisat, A. M. S., Tableb, O. K., Al-Mugheed, K., Alabdullah, A. A., & Abdelaliem, S. M. F. (2024). The impact of a nursing spiritual care module on nursing competence: An experimental design. *BMC Palliative Care,**23*(1), 21. 10.1186/s12904-024-01356-z 38246991 10.1186/s12904-024-01356-z PMC10802070

[CR3] Alch, C. K., Wright, C. L., Collier, K. M., & Choi, P. J. (2021). Barriers to addressing the spiritual and religious needs of patients and families in the intensive care unit: A qualitative study of critical care physicians. *The American Journal of Hospice and Palliative Care,**38*(9), 1120–1125. 10.1177/1049909120970903 33143446 10.1177/1049909120970903

[CR4] Aslan, H., Aktürk, Ü., & Erci, B. (2020). Validity and reliability of the Turkish version of the nurse spiritual care therapeutics scale. *Palliative and Supportive Care,**18*(6), 707–712. 10.1017/S1478951520000267 32390585 10.1017/S1478951520000267

[CR5] Badanta, B., Rivilla-García, E., Lucchetti, G., & de Diego-Cordero, R. (2022). The influence of spirituality and religion on critical care nursing: An integrative review. *Nursing in Critical Care,**27*(3), 348–366. 10.1111/nicc.12645 33966310 10.1111/nicc.12645

[CR6] Baguna, A. E., Pandeirot, C. Y. M., Juniarta, & Barus, N. S. (2024). Correlation of nurses’ perception of spirituality and spiritual care with spiritual care practices in Indonesia: A cross-sectional survey. *Belitung Nursing Journal,**10*(5), 593–600. 10.33546/bnj.3467 39416356 10.33546/bnj.3467 PMC11474262

[CR7] Bakir, E., Samancioglu, S., & Kilic, S. P. (2017). Spiritual experiences of muslim critical care nurses. *Journal of Religion and Health,**56*(6), 2118–2128. 10.1007/s10943-017-0382-4 28342145 10.1007/s10943-017-0382-4

[CR8] Berning, J. N., Poor, A. D., Buckley, S. M., Patel, K. R., Lederer, D. J., Goldstein, N. E., Brodie, D., & Baldwin, M. R. (2016). A novel picture guide to improve spiritual care and reduce anxiety in mechanically ventilated adults in the intensive care unit. *Annals of the American Thoracic Society,**13*(8), 1333–1342. 10.1513/AnnalsATS.201512-831OC 27097049 10.1513/AnnalsATS.201512-831OC PMC5021077

[CR9] Bone, N., Swinton, M., Hoad, N., Toledo, F., & Cook, D. (2018). Critical care nurses’ experiences with spiritual care: The SPIRIT Study. *American Journal of Critical Care,**27*(3), 212–219. 10.4037/ajcc2018300 29716908 10.4037/ajcc2018300

[CR10] Çınar, F., & Aslan, F. E. (2017). Spiritualism and nursing: the importance of spiritual care in intensive care patients. *Journal of Academic Research in Nursing,**3*(1), 37–42. 10.5222/jaren.2017.037

[CR11] Daghan, S., Kalkim, A., & Sağkal Midilli, T. (2019). Psychometric evaluation of the Turkish form of the spiritual care competence scale. *Journal of Religion and Health,**58*(1), 14–27. 10.1007/s10943-018-0594-2 29524070 10.1007/s10943-018-0594-2

[CR12] Dalle Ave, A. L., & Sulmasy, D. P. (2025). Does sedation affect patients’ spiritual experience at the end of life? An intersection between medicine and spirituality. *Journal of Pain and Symptom Management,**69*(1), e86–e89. 10.1016/j.jpainsymman.2023.10.023 37871840 10.1016/j.jpainsymman.2023.10.023

[CR13] Dural, G. (2024). Spiritual care experiences of nurses working in intensive care units: A qualitative study. *Nursing in Critical Care,**29*(3), 545–554. 10.1111/nicc.12975 37667443 10.1111/nicc.12975

[CR14] Harrad, R., Cosentino, C., Keasley, R., & Sulla, F. (2019). Spiritual care in nursing: an overview of the measures used to assess spiritual care provision and related factors amongst nurses. *Acta Bio-Medica,**90*(4-S), 44–55. 10.23750/abm.v90i4-S.8300 30977748 10.23750/abm.v90i4-S.8300 PMC6625560

[CR15] Heidari, A., Afzoon, Z., & Heidari, M. (2022). The correlation between spiritual care competence and spiritual health among Iranian nurses. *BMC Nursing,**21*(1), 277. 10.1186/s12912-022-01056-0 36224620 10.1186/s12912-022-01056-0 PMC9555262

[CR16] Hughes, B., Whitmer, M., & Hurst, S. (2007). Innovative solutions: A plurality of vision–integrating the chaplain into the critical care unit. *Dimensions of Critical Care Nursing,**26*(3), 91–95. 10.1097/01.DCC.0000267801.62949.6d 17440290 10.1097/01.DCC.0000267801.62949.6d

[CR17] Kappes, M., Fernández-Silva, C. A., Catalán, L., Navalle, C., Diaz, M., & Guglielmi, I. (2025). Nurses’ role in spiritual care for patients and families in intensive care units: A scoping review. *Enfermeria Intensiva,**36*(1), Article 100494. 10.1016/j.enfie.2025.100494 39827495 10.1016/j.enfie.2025.100494

[CR18] Klimasiński, M. W. (2021). Spiritual care in the intensive care unit. *Anaesthesiology Intensive Therapy,**53*(4), 350–357. 10.5114/ait.2021.109920 34714016 10.5114/ait.2021.109920 PMC10165982

[CR19] Koroglu, S., & Öksüz, E. (2024). How emotional contagion shapes spiritual care competence: Insights from a cross-sectional study on intensive care nurses. *Nursing in Critical Care,**29*(6), 1394–1404. 10.1111/nicc.13160 39318081 10.1111/nicc.13160

[CR20] Kurtgöz, A., Keten Edis, E., & Erarslan, R. (2024). Spiritual care competencies and the frequency of spiritual care practices of nurses in Turkey. *Journal of Religion and Health,**63*(3), 1747–1760. 10.1007/s10943-023-01884-7 37540306 10.1007/s10943-023-01884-7

[CR21] Li, L., Chen, M., Yu, N., & Zhang, Q. (2024). Effectiveness of spiritual care interventions among patients in the intensive care unit: A systematic review and meta-analysis. *Nursing in Critical Care*. Advance Online Publication. 10.1111/nicc.13202 10.1111/nicc.13202 39467701

[CR22] Mamier, I., & Taylor, E. J. (2015). Psychometric evaluation of the nurse spiritual care therapeutics scale. *Western Journal of Nursing Research,**37*(5), 679–694. 10.1177/0193945914530191 24718038 10.1177/0193945914530191

[CR23] Meeprasertsagool, N., Anuraktham, P., Chaithanasarn, A., & Wongprom, I. (2025). Future directions of spiritual care where spiritual care providers do not exist: A qualitative study. *BMC Palliative Care,**24*(1), 19. 10.1186/s12904-025-01658-w 39833758 10.1186/s12904-025-01658-w PMC11744905

[CR24] Mohamed Elsayed, S., Abdalla Elbiaa, M., & Arafa Hassan, E. (2023). Critical care nurses’ spiritual care practice and its relationship with their spiritual perception and competency. *Alexandria Scientific Nursing Journal,**25*(4), 64–73. 10.21608/asalexu.2023.344753

[CR25] Momeni, G., Hashemi, M. S., & Hemati, Z. (2022). Barriers to providing spiritual care from a nurses’ perspective: A content analysis study. *Iranian Journal of Nursing and Midwifery Research,**27*(6), 575–580. 10.4103/ijnmr.ijnmr_422_21 36712312 10.4103/ijnmr.ijnmr_422_21 PMC9881555

[CR26] Riahi, S., Goudarzi, F., Hasanvand, S., Abdollahzadeh, H., Ebrahimzadeh, F., & Dadvari, Z. (2018). Assessing the effect of spiritual intelligence training on spiritual care competency in critical care nurses. *Journal of Medicine and Life,**11*(4), 346–354. 10.25122/jml-2018-0056 30894893 10.25122/jml-2018-0056 PMC6418341

[CR27] Timmins, F., & Caldeira, S. (2017). Understanding spirituality and spiritual care in nursing. *Nursing Standard,**31*(22), 50–57. 10.7748/ns.2017.e10311 28120672 10.7748/ns.2017.e10311

[CR28] Van Leeuwen, R., Tiesinga, L. J., Middel, B., Post, D., & Jochemsen, H. (2009). The validity and reliability of an instrument to assess nursing competencies in spiritual care. *Journal of Clinical Nursing,**18*, 2857–2869. 10.1111/j.1365-2702.2008.02594.x 19220618 10.1111/j.1365-2702.2008.02594.x

[CR29] Vogel, A., & Schep-Akkerman, A. E. (2018). Competence and frequency of provision of spiritual care by nurses in the Netherlands. *Scandinavian Journal of Caring Sciences,**32*(4), 1314–1321. 10.1111/scs.12575 29691885 10.1111/scs.12575

[CR30] Wall, R. J., Engelberg, R. A., Gries, C. J., Glavan, B., & Curtis, J. R. (2007). Spiritual care of families in the intensive care unit. *Critical Care Medicine,**35*(4), 1084–1090. 10.1097/01.CCM.0000259382.36414.06 17334245 10.1097/01.CCM.0000259382.36414.06

[CR31] Willemse, S., Smeets, W., van Leeuwen, E., Janssen, L., & Foudraine, N. (2018). Spiritual care in the ICU: Perspectives of dutch intensivists, ICU nurses, and spiritual caregivers. *Journal of Religion and Health,**57*(2), 583–595. 10.1007/s10943-017-0457-2 28801715 10.1007/s10943-017-0457-2 PMC5854753

[CR32] Willemse, S., Smeets, W., van Leeuwen, E., Nielen-Rosier, T., Janssen, L., & Foudraine, N. (2020). Spiritual care in the intensive care unit: An integrative literature research. *Journal of Critical Care,**57*, 55–78. 10.1016/j.jcrc.2020.01.026 32062288 10.1016/j.jcrc.2020.01.026

